# Risk factors for spontaneous abortion following hepatitis E vaccination during and shortly before pregnancy: Further analysis from a cluster-randomized trial

**DOI:** 10.1371/journal.pone.0345974

**Published:** 2026-04-10

**Authors:** Asma Binte Aziz, Faisal Ahmmed, Kassa Haile, Kelvin Kering, Susanne Dudman, Cathinka Halle Julin, Joakim Øverbø, Ashraful Islam Khan, Afroza Akter, Beatrice Atieno Ongadi, Benjamin Ngugi, Cecilia Kathure Mbae, Edlawit Mesfin, Fahima Chowdhury, Farhana Khanam, Joo-Young Esther Lee, Justin Im, Martin Mwebia Bundi, Md Golam Firoj, Md Taufiqul Islam, Moses Mwangi, Natasha Y. Rickett, Sadia Isfat Ara, Meseret Gebre Behute, Se Eun Park, Suneth Agampodi, Samuel Kariuki, Suman Kanungo, Deok Ryun Kim, Firdausi Qadri, Khalequ Zaman, John D Clemens

**Affiliations:** 1 International Vaccine Institute, Seoul, Republic of Korea; 2 International Centre for Diarrhoeal Disease Research, Dhaka, Bangladesh; 3 Institute of Clinical Medicine, University of Oslo, Norway; 4 Armauer Hansen Research Institute, Addis Ababa, Ethiopia; 5 Kenya Medical Research Institute (KEMRI), Nairobi, Kenya; 6 Oslo University Hospital, Oslo, Norway; 7 Research Investment for Global Health Technology (RIGHT) Foundation, Seoul, Republic of Korea; 8 ICMR - National Institute of Cholera and Enteric Diseases, Kolkata, West Bengal, India; 9 UCLA Fielding School of Public Health, Los Angeles, California, United States of America; The University of New Mexico, UNITED STATES OF AMERICA

## Abstract

**Background:**

A recent randomized trial of Hepatitis E Vaccine (HEV239) found an elevated risk of spontaneous abortion (SAB) in women given HEV239 during or within ninety days before pregnancy. Here we present additional analyses to better understand this risk.

**Methods:**

We investigated whether maternal risk profiles of SAB or gestational age at SAB differed between women receiving HEV239 versus control Hepatitis B vaccine (HBV). We also assessed whether HEV239-vaccinated women who experienced SABs had distinct short-term adverse reactions compared to HEV239-vaccinated women who had live births, exploring potential inflammatory mechanisms. Finally, we assessed maternal exposure to HEV239 in narrowly defined pre-last menstrual period (LMP) windows to more precisely demarcate pre-LMP at which HEV-239 vaccination was associated with an elevated risk of SAB. Chi-square tests and Mann-Whitney U tests were performed for bivariate comparisons. Poisson models were used for multivariable analyses.

**Findings:**

Baseline maternal risk factors for SAB (age, body mass index, parity, and histories of SAB, hypertension, or diabetes) were similar between the SABs in HEV239 and HBV groups. Median gestational age at SAB was comparable (10.5 weeks [IQR 10-13] for HEV239 vs. 11 weeks [IQR 9-12.2] for HBV, p = 0.783). No increased short-term adverse events (pain: 9% vs. 9%; fever: 2% vs. 1%) were observed among HEV239 recipients who experienced SABs compared to those who did not. An elevated risk of SAB was observed in HEV239 recipients vaccinated 31–60 days pre-LMP (Relative Risk 4.3 [95% CI 1.3–19.3], p = 0.026).

**Interpretation:**

Our analyses did not uncover associations pointing to a biological mechanism underlying the original observation of an increased SAB rate connected to HEV239 vaccination shortly before or during pregnancy. Targeted studies such as placental examinations and genetic testing are needed to investigate potential mechanisms.

## Introduction

Pregnant women are at the highest risk of life-threatening complications from hepatitis E infection, followed by immunocompromised individuals and those with chronic liver disease [1]. To address the needs of these high-risk populations, the recombinant hepatitis E vaccine (HEV239) was developed for use during hepatitis E outbreaks [2]. While HEV239 has demonstrated excellent safety and efficacy in the general population [3,4], research involving pregnant women remains limited, leaving a crucial gap in our understanding of its safety for this group. A limited post hoc analysis, involving a small number of women inadvertently vaccinated during pregnancy, showed no indications of fetal risk [5,6]. Further, an observational study following a large-scale vaccination campaign in South Sudan also found no increased risk of fetal loss among pregnant women [7].

However, recent results from our double-blind, cluster-randomized trial conducted in rural Bangladesh raised new questions about the safety of HEV239 for pregnant women. The study indicated that women who were administered HEV239 during pregnancy or within ninety days before pregnancy a pre specified interval also used in other analyses of this topic [5] demonstrated a two-fold increased risk of spontaneous abortion (SAB) compared to those receiving a control hepatitis B vaccine (HBV) [8]. Among women vaccinated during pregnancy, most within the first 30 days after LMP, HEV239 recipients had a significantly higher risk of SAB compared with HBV recipients (adjusted RR 2.1; 95% CI 1.1–4.1; p = 0.036) [8].

In light of these findings, we conducted an extended analysis of the trial to uncover possible clues to the etiology of the elevated risk observed in HEV-vaccinated women. In this paper we report whether maternal risk factors for SAB and gestational age at SAB differed among women who experienced SAB in HEV239 versus HBV groups. We also assessed whether HEV239-vaccinated women who experienced SAB had enhanced short-term adverse reactions compared with HEV239-vaccinated women who did not experience SAB. Furthermore, we examined HEV239 vaccination timing in relation to pregnancy and SAB risk in narrower time intervals during the pre- pregnancy period.

## Methods

### Study participants and procedure

This analysis was performed on data from a cluster-randomized, double-blind, controlled phase IV study conducted between October 2017 and October 2021 in rural Bangladesh. The primary objective was to assess the HEV239 vaccine’s effectiveness among women of childbearing age [9]. In total, 67 clusters (villages) were randomized, with 33 clusters assigned to receive the HEV239 vaccine and 34 clusters to the control HBV vaccine, administered at 0, 1, and 6 months. The trial recruited 19,460 non-pregnant women aged 16–39 years, and each participant was closely monitored for 30 minutes following vaccination. They were then followed for 7 days through home visits after each dose with additional follow-ups for two years post-final dose to record any adverse events, detect pregnancies, and document pregnancy outcomes.

To ensure that pregnancy status was accurately assessed, all women were screened with a high-sensitivity urine test [10,11] before each dose if they reported a missed period, irregular cycles, or uncertainty about their last menstrual period (LMP). Pregnant women were excluded from further vaccination; however, any pregnancies detected after the first or second dose were followed bi-weekly until the end of pregnancy. Detailed procedures for pregnancy detection, follow-up, and outcome diagnosis are available elsewhere [8].

For the present risk factor analyses, we focused specifically on a subset of pregnant women who had received either the HEV239 or HBV vaccine during the “proximal period,” (defined *a priori* as the interval of ninety days before LMP through the end of pregnancy) and who subsequently experienced SAB. SABs were diagnosed by study physicians and verified via ultrasound, with prospective data collection of baseline risk factors related to SAB and pregnancy outcomes. SAB was defined following WHO guidelines as pregnancy loss happening before 20 weeks of gestation with no human intervention. Stillbirth was defined as fetal loss at 20 weeks of gestation or later (≥140 days). Induced abortion was specified as the deliberate termination of pregnancy through a medical or surgical procedure [8]. Baseline maternal risk factors including body mass index (BMI), age, history of SAB, hypertension, and diabetes data were collected as part of the protocol. The gestational age at SAB was determined based on the date of LMP and pregnancy outcome date as determined by study physicians and verified via ultrasound.

The trial protocol received approval from the Ethics Committee of the icddr,b, the Directorate General of Drug Administration (DGDA) Bangladesh, and the Ethics Committee of Oslo, Norway. Additionally, it was reviewed by the Norwegian Institute of Public Health. An independent data and safety monitoring board, established by icddr,b, oversaw participant’s safety and progress of the project. All participants provided informed written consent. The trial was registered on ClinicalTrials.gov under the identifier NCT02759991.

### Statistical analysis

As in the original analysis, the dose administered closest to the LMP date was considered the most biologically relevant for analyzing the effect of vaccination on pregnancy outcomes. We classified the date of this dose as zero time and used zero time to determine the temporal relationship between vaccination and the LMP.

To investigate maternal risk factors that might influence SAB risk, we analyzed baseline characteristics, determined at the time of entry of women into the trial, among women who developed SABs after receiving HEV239 and HBV vaccines. We also compared gestational ages at the time of SAB between the HEV239 and HBV groups to assess any potential biological differences. We employed two approaches for both comparisons. First, we compared the baseline risk factors among women who experienced SAB in HEV239 group to those in HBV group in women vaccinated during the proximal period. In the second approach, to increase the sample size, we compared SABs in the proximal period for HEV to SABs occurring in both proximal and distal (defined as more than ninety days before the LMP) for the HBV group. We also assessed whether vaccine adverse reactions within seven days of zero time were more common among women who experienced SAB than among those with live births, among HEV239 recipients in the proximal period. The adverse events analyzed included local pain, swelling, itching, redness and systemic symptoms including fever, headache, nausea/vomiting, asthenia/fatigue, myalgia, allergies, and malaise. For these bivariate analyses, categorical variables were compared with the Chi-square test, or with Fisher's exact test when data were sparse, while continuous variables were compared using the Student's t-test, or the Mann–Whitney U test when data were not normally distributed.

To more precisely capture the relationship between the timing of pre-LMP receipt of HEV239 and elevation of risk of SAB, we analyzed and refined the pre-LMP risk period by examining whether SAB risk varied for zero times occurring in the following six intervals before the LMP: 0–30, 31–60, 61–90, 91–120, 121–150, and 151–180 days. For each of these intervals, we compared the baseline variables affecting the risk for SAB among women receiving HEV239 and HBV vaccines. We estimated crude relative risks (RR) of SAB for women vaccinated with HEV239 vs. HBV in each interval, using Poisson models in the same manner as the original analysis with vaccine status as the independent variable and SAB as the dependent variable. As no risk factors were significantly associated with the risk of SAB (p < 0.05), no additional covariates were included. The coefficient for the vaccine variable was exponentiated to estimate the relative risk and the standard error of the coefficient was used to estimate the 95% CI and p-value. We did not perform multivariable logistic regression models including multiple covariates simultaneously for several reasons. First, the bivariate analyses revealed no maternal risk factors that were significantly associated with SAB risk in HEV239 recipients, suggesting that traditional risk factors did not explain the observed vaccine-associated risk. Second, given the limited number of SAB events in certain subgroups (particularly in the narrowly-defined pre-LMP time intervals), multivariable models would risk overfitting and produce unstable estimates. The exploratory nature of these analyses, particularly the refined temporal assessments, prioritized identifying potential risk windows rather than generating adjusted risk estimates. Where relevant, we used Poisson models to estimate relative risks adjusted for the clustering design, which was appropriate given the study design and event distribution.

We additionally conducted a more granular assessment of the timing effect using a continuous-time framework for the pre-LMP period. Specifically, we fit an interaction model including a restricted cubic spline term (2 degrees of freedom) for time from vaccination to LMP and an interaction between the spline and vaccine assignment. This model was used to estimate the weekly predicted risk of SAB and the corresponding risk ratios with 95% confidence intervals.

Statistical analyses were conducted using STATA 15 and R (version 4.2.1), with statistical significance defined as a two-tailed p-value below 0.05.

## Results

From October 2017 to October 2021, a total of 19,460 women, aged 16–39, who were classified as not pregnant at the time, were enrolled in the study across 67 clusters. Among them, 5,011 women became pregnant during the study period. Of these, 1,450 were inadvertently vaccinated during the proximal period. Within this group, 209 received HEV239 during pregnancy and 398 shortly before pregnancy. A total of 86 SABs were reported among the 1,450 women, with the majority (63%, 54/86) occurring in those who received HEV239 [8].

### Risk factor analysis for SAB in HEV239 vaccine recipients

We compared standard baseline characteristics that could potentially affect the SAB risk between women who had SAB in the HEV239 and HBV groups after being dosed in the proximal period. The median age at the time of the first positive pregnancy test was 25 years (IQR 19–32) in the HEV239 group and 26 years (IQR 21–30) in the HBV group (p = 0.549). Similarly, the median gestational age at SAB was 10.5 weeks (IQR 10–13) for HEV239 and 11 weeks (IQR 9–12.2) for HBV group (p = 0.783) at the time of SAB. The mean BMI at enrollment was 23.2 (IQR 20.4–26.3) in the HEV239 group and 25.0 (IQR 20.9–28.6) in the HBV group (p = 0.209). History of SAB was reported in 11 women (20%) in the HEV239 group and 8 women (25%) in the HBV group (p = 0.617). There were no significant differences between the HEV239 and HBV groups in any of the assessed risk factors (Table 1).

**Table 1:** Baseline risk factors for spontaneous abortion (SAB) in the HEV239 and HBV vaccine groups dosed during the proximal period.

Characteristic	SAB in HEV239 group, N = 54^1^	SAB in HBV group, N = 32^1^	p-value^2^
**Maternal age at 1st pregnancy test (Median, IQ Range)**	25 (19, 32)	26 (21, 30)	0.549
**Maternal age group at 1st pregnancy test**			0.191
16-19, years	17 (31.5%)	5 (15.6%)	
20-35, years	30 (55.6%)	24 (75.0%)	
36-40, years	7 (13.0%)	3 (9.4%)	
**Gestational age at SAB, weeks (Median, IQ Range)**	10.5 (10, 13)	11 (9, 12.2)	0.783
**Gestational age group at SAB**			0.862
0-3, weeks	1 (1.9%)	0 (0.0%)	
4-7, weeks	3 (5.6%)	3 (9.4%)	
8-11, weeks	28 (51.9%)	15 (46.9%)	
12-19, weeks	22 (40.7%)	14 (43.8%)	
**Gestational age at first positive pregnancy test, weeks (Median, IQ Range)**	8 (7, 9)	7 (6, 9)	0.416
**Gestational age group at first positive pregnancy test**			0.426
0-3, weeks	2 (3.7%)	0 (0.0%)	
4-7, weeks	22 (40.7%)	17 (53.1%)	
8-11, weeks	26 (48.1%)	11 (34.4%)	
12-19, weeks	4 (7.4%)	4 (12.5%)	
**BMI at enrollment (Median, IQ Range)**	23.2 (20.4, 26.3)	25.0 (20.9, 28.6)	0.209
**BMI group at enrollment (dose 1)**			0.142
<=30	51 (94.4%)	27 (84.4%)	
>30	3 (5.6%)	5 (15.6%)	
**History of SAB**			0.617
Yes	11 (20.4%)	8 (25.0%)	
No	43 (79.6%)	24 (75.0%)	
**History of induced /therapeutic abortion**			0.666
Yes	3 (5.6%)	3 (9.4%)	
No	51 (94.4%)	29 (90.6%)	
**History of Hypertension**			0.788
Yes	2 (3.7%)	1 (3.1%)	
No	50 (92.6%)	31 (96.9%)	
**Parity**			0.196
0	16 (29.6%)	5 (15.6%)	
>=1	38 (70.4%)	27 (84.4%)	
**History of stillbirth**			0.356
Yes	2 (3.7%)	3 (9.4%)	
No	52 (96.3%)	29 (90.6%)	
**History of Diabetes**			0.527
Yes	0 (0.0%)	0 (0.0%)	
No	54 (100.0%)	32 (100.0%)	

^1^ n (%); Median (IQR)

^2^ Fisher's exact test; Pearson's Chi-squared test; Wilcoxon rank sum test

As a second approach, we also compared the 54 women in the HEV239 group who had SABs after receiving doses during the proximal period with 112 women in the HBV group who had a SAB after receiving doses during either the proximal or distal periods. This comparison yielded findings consistent with our primary analysis, showing no statistically significant differences in any of the assessed risk factors for SAB (Table 2).

**Table 2:** Baseline risk factors for spontaneous abortion (SAB) in the HEV239 groups dosed during the proximal period and the HBV group dosed during both the proximal and distal periods.

Characteristic	SAB in HEV group, N = 54^1^	SAB in HBV group, N = 112^1^	p-value^2^
**Maternal age at 1st pregnancy test (Median, IQ Range)**	25 (19, 32)	24 (21, 31)	0.542
**Maternal age group at 1st positive pregnancy test**			0.114
16-19, years	17 (31.5%)	20 (17.9%)	
20-35, years	30 (55.6%)	79 (70.5%)	
36-40, years	7 (13.0%)	13 (11.6%)	
**Gestational age at SAB (Median, IQ Range)**	10.5 (10, 13)	12 (9, 14)	0.692
**Gestational age group SAB**			0.098
0-3, weeks	1 (1.9%)	0 (0.0%)	
4-7, weeks	3 (5.6%)	13 (11.6%)	
8-11, weeks	28 (51.9%)	42 (37.5%)	
12-19, weeks	22 (40.7%)	57 (50.9%)	
**Gestational age at first positive pregnancy test (Median, IQ Range)**	8 (7, 9)	7 (6, 9)	0.354
**Gestational age group at first positive pregnancy test**			0.089
0-3, weeks	2 (3.7%)	0 (0.0%)	
4-7, weeks	22 (40.7%)	61 (54.5%)	
8-11, weeks	26 (48.1%)	42 (37.5%)	
12-19, weeks	4 (7.4%)	9 (8.0%)	
**BMI at enrollment (Median, IQ Range)**	23.2 (20.4, 26.3)	23.1 (20.2, 26.0)	0.808
**BMI group at enrollment (dose 1)**			>0.999
<=30	51 (94.4%)	104 (92.9%)	
>30	3 (5.6%)	8 (7.1%)	
**History of SAB**			0.403
Yes	11 (20.4%)	17 (15.2%)	
No	43 (79.6%)	95 (84.8%)	
**History of induced /therapeutic abortion**			>0.999
Yes	3 (5.6%)	7 (6.2%)	
No	51 (94.4%)	105 (93.8%)	
**History of Hypertension in pregnancy**			0.184
Yes	2 (3.7%)	4 (3.6%)	
No	50 (92.6%)	108 (96.4%)	
Unknown	2 (3.7%)	0 (0.0%)	
**Parity**			0.714
0	16 (29.6%)	30 (26.8%)	
>=1	38 (70.4%)	82 (73.2%)	
**History of stillbirth**			>0.999
Yes	2 (3.7%)	6 (5.4%)	
No	52 (96.3%)	106 (94.6%)	
**History of Diabetes**			0.104
Yes	0 (0.0%)	1 (0.9%)	
No	54 (100.0%)	111 (99.1%)	

^1^ n (%); Median (IQR)

^2^ Fisher's exact test; Pearson's Chi-squared test; Wilcoxon rank sum test

### Comparison of immediate adverse events in HEV239 recipients

We compared immediate adverse events (AEs) reported within 7 days of any dose between 54 women who experienced SAB and 599 women with live births after receiving HEV239 during the proximal period. Few local and systemic AEs were reported, and there were no significant differences between women who experienced SAB versus those who did not. Pain was the most common side effect, reported by 5 women (9%) who had SAB and 54 (9%) who had live birth, followed by fever, with 1 (2%) among women who had SAB and 6 (1%) who had the live birth (Table 3).

**Table 3:** Adverse events among women who had a spontaneous abortion (SAB) and those who had a live birth in HEV239 group vaccinated during the proximal period.

Adverse events within 7 days after vaccination	Women who had SAB N = 54	Women who had live birth N = 599	p-value
**Local**			
Pain	5 (9.3)	54 (9.0)	>0.999
Swelling or induration	0 (0.0)	1 (0.2)	
Itching	0 (0.0)	0 (0.0)	
Redness	0 (0.0)	0 (0.0)	
**Systemic**			
Fever	1 (1.9)	6 (1.0)	
Headache	0 (0.0)	3 (0.5)	
Nausea or vomiting	0 (0.0)	1 (0.2)	
Asthenia or fatigue	0 (0.0)	1 (0.2)	
Myalgia	0 (0.0)	0 (0.0)	
Allergic reaction	0 (0.0)	0 (0.0)	
Malaise	0 (0.0)	0 (0.0)	

### Comparison of SAB risk between HEV239 and HBV recipients before pregnancy

Baseline characteristics potentially affecting the SAB risk were consistently comparable between HEV239 and HBV recipients across all periods before LMP at intervals of LMP-30 days, 31–60 days, 61–90 days, 91–120 days, 121–150 days, and 151–180 days. None of the assessed risk factors for SAB during these six periods showed statistically significant differences (S1–S6 Tables).

**Fig 1:**
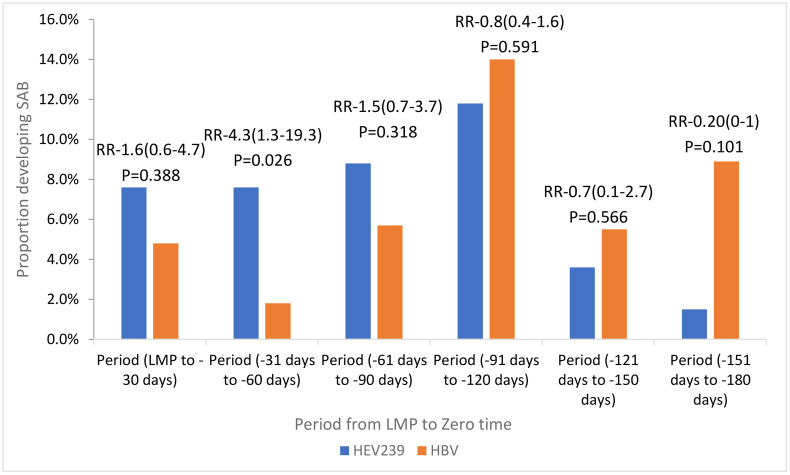
Association between vaccination and spontaneous abortion (SAB) among women whose zero-time occurred from LMP to −180 days in 1-month intervals.

Significantly elevated point estimates were observed only among women with zero times during the 31–60 days prior to LMP, with no significant differences in earlier intervals. During the period from LMPto -30 days, 9 (8%) of 119 women receiving HEV239 and 6 (5%) of 125 women who received HBV experienced SABs (crude RR 1.6 [95% CI 0.6–4.7], p = 0.388). In the LMP-31 to -60 days period, SABs occurred in 10 (8%) of 131 women in the HEV239 group and 3 (2%) of 170 in the HBV group, with a significantly elevated crude RR of 4.3 ([95% CI 1.3–19.3], p = 0.026). For earlier intervals (LMP-61 to -180 days), no significant variations in SAB risk were observed between the groups (Fig 1).

Using the spline-based interaction model, we estimated the weekly risk of SAB across the full pre-LMP period (S1 Fig). The results show that the elevated SAB risk associated with HEV239 is concentrated within approximately the first 8 weeks before LMP. Beyond this window, the predicted risks in the two groups converge and the risk ratio is no longer statistically significant.

## Discussion

In this extended analysis, we set out to determine if certain patterns in maternal risk factors, SAB characteristics, or timing of vaccination could provide clues to the mechanism behind the increased risk of SAB in HEV239 vaccine recipients observed in the original trial [8]. We found that baseline risk factors associated with SABs— such as maternal age, BMI, and histories of SAB, hypertension, or diabetes [12,13]—were similarly distributed between the HEV239 and HBV groups, suggesting that maternal characteristics are unlikely to be driving the elevated risk of SABs in HEV239 recipients. Additionally, the gestational age at the time of SAB was similar between the two groups, suggesting that HEV239 did not accelerate the timing of SAB. We also found no evidence of enhanced short-term adverse events following HEV239 vaccination that could suggest a possible inflammatory mechanism for the observed elevation of SAB risk in HEV239 recipients. However, our analysis did reveal that SAB risk was higher in the HEV239 recipients during a critical window, specifically up to 60 days before the LMP, with no excess risk observed beyond this period. This finding suggests that exposure closer to LMP may increase SAB risk, highlighting a potential time-dependent aspect that warrants further investigation.

The absence of a known biological basis for an increase in the risk of SAB observed in our study in no way diminishes the credibility of our findings. Established guidelines for evaluating epidemiological evidence recognize that pinpointing a precise biological mechanism is not essential for validating the findings from well-designed and conducted studies, and there are numerous examples of well-accepted vaccine side effects whose biological basis is unknown or poorly understood [14,15]. Furthermore, our observation that HEV239 exposure was only associated with an elevated risk of SAB for the 60 days immediately preceding the LMP aligns with what might be expected if there were an actual effect related to timing of vaccination relative to conception.

Recent experimental models suggest that immune responses, particularly the activation of inflammatory pathways by virus-like particles (VLPs) in the hepatitis E Virus [16], may underlie the observed elevated risk of SAB during the proximal period [17]. This hypothesis should be evaluated in future human studies, ideally with a detailed analysis of placentas and fetuses from HEV239-exposed women who experience SABs [17,18].

A large-scale vaccination campaign during an HEV epidemic in South Sudan, which included more than 2,000 pregnant women, showed no increased risk of SAB associated with HEV239 [7]. However, several limitations exist that require careful consideration when interpreting this result. The study relied heavily on self-reported data, and approximately 40% of women did not have records to confirm their vaccination dates and the onset of pregnancy. Moreover, pregnancies were often not confirmed, abortion diagnoses were sometimes inaccurate, and vaccine histories were incomplete. Moreover, the protective effect of the HEV vaccination against HEV infection may have weakened the ability of the study to assess the link between receiving the vaccine and experiencing SAB, since HEV infection itself is a cause of SAB. Our study’s prospective design with weekly pregnancy surveillance, biweekly follow-up until pregnancy outcome and ultrasound confirmation of SABs, coupled with independent validation by the Matlab Maternal and Child Health (MCH) program, minimized the risk of reporting or misclassification bias. Additionally, very little HEV infection occurred during the trial, increasing the trial’s ability to unmask the HEV vaccine-SAB relationship.

We acknowledge that our ability to accurately assess histories of multiple prior SABs was limited, as the magnitude of risk has been shown to increase with the number of prior losses [19]. However, participants were relatively young (16–39 years old), and very few had more than one previous SAB. Among women vaccinated during the proximal period, only about 20–25% had any history of SAB. Consequently, we categorized this variable as binary (≥1 prior SAB vs. none), which may have reduced our ability to detect dose–response effects related to the number of previous losses. Additionally, while some clinical causes of SAB that are routinely evaluated in U.S. settings—such as syphilis infection—were not systematically assessed in our study, syphilis prevalence among women of reproductive age in Bangladesh is extremely low. Therefore, it is unlikely that untreated syphilis or similar rare infections meaningfully contributed to the observed difference in SAB risk between vaccine groups.

Regarding statistical interpretation, we note that journal reviewers sometimes request post-hoc power calculations to assess the adequacy of sample size for subgroup analyses that yield nonsignificant results. However, such calculations are theoretically unsound and represent a fundamental misconception that persists in medical literature despite extensive documentation by statisticians [20–24]. Statistical power is defined as the probability of obtaining a statistically significant result in a future study under specified conditions; once data are collected and results are known, this probability concept becomes meaningless [21,22]. As Goodman and Berlin [21] state explicitly, “power should play no role once the data have been collected.” Moreover, for any nonsignificant result, post-hoc power will always be less than 50% and is mathematically determined by the p-value itself, providing no additional information beyond what the p-value already conveys [20,21]. The appropriate method for assessing whether nonsignificant findings might represent false negatives is to examine confidence intervals for effect estimates [21,22]. Wide confidence intervals indicate limited precision and suggest that clinically meaningful associations cannot be definitively ruled out, while narrow intervals excluding meaningful effects provide reassurance against important missed associations. We have provided 95% confidence intervals for all effect estimates throughout this manuscript, allowing readers to assess the precision of our findings and judge which effect sizes remain plausible. For subgroup analyses with limited events (such as the narrowly-defined pre-LMP intervals), the wide confidence intervals appropriately reflect the uncertainty in these exploratory assessments, and null findings should be interpreted cautiously as they may reflect limited sample size rather than definitive absence of association.

It is important to interpret our findings in the context of severe maternal outcomes from HEV infection. While the absolute risk increase of SAB associated with HEV239 is modest (~5%), we believe it is important not to downplay the two-fold elevated risk observed during early pregnancy, particularly within 30 days of conception [8]. This is directly relevant because HEV239 is intended for use in outbreak settings, where pregnancy screening may not be feasible and women may be unknowingly pregnant at the time of vaccination. Reporting both the relative and absolute risk in the original paper ensures a transparent and balanced interpretation of the findings, providing critical information for policymakers and clinicians to weigh the risk of early pregnancy loss against the risk of maternal mortality from HEV (~20%). Importantly, the elevated SAB risk applies to all women who are vaccinated during early pregnancy, whereas the risk of maternal mortality applies only to women who become infected with HEV. The latter varies substantially by setting—from very low risk in non-outbreak environments like rural Matlab, where our study was conducted, to very high risk in refugee settings experiencing active HEV transmission. For this reason, presenting both risks clearly is essential to support informed decision-making across diverse epidemiological contexts. As a precaution, policymakers and clinicians may consider advising women who are planning pregnancy to wait about one month after vaccination before attempting conception, given that the time-linked SAB risk was observed both shortly after vaccination and during the 60-day pre-LMP period.

In conclusion, while our analyses do not provide clues to an underlying mechanism, they do not refute the initial epidemiological findings. They instead underscore the need for more targeted biological data, such as placental examinations and genetic testing of products of conception. Although our exploration of biological mechanisms was necessarily limited, this gap highlights a critical direction for future research—particularly to clarify whether immune-mediated or inflammatory responses to HEV239 might explain the observed time-dependent risk. Future studies should focus on exploring immune-mediated changes [17], particularly in aborted fetuses or placental tissues, to better understand potential mechanisms.

## Research in context

### Evidence before this study

We searched through the PubMed and pre-print platforms until November 25, 2024, using the search terms (“HEV vaccination” OR “HEV239 vaccine”) AND (“pregnancy”) AND (“spontaneous abortion” OR “miscarriage”) AND (“risk factors”). Our search included randomized controlled trials, observational studies, and meta-analyses reporting on pregnancy outcomes following HEV239 vaccination. We did not identify any studies that specifically investigated risk factors for spontaneous abortion in HEV239-vaccinated pregnant women. We found only three studies examining the relationship between HEV239 vaccination and pregnancy outcomes, including miscarriage or spontaneous abortion risk. A post hoc analysis of a Phase 3 trial in China involving only 66 women inadvertently vaccinated with HEV239 during pregnancy showed no indications of fetal risk. An observational study in Bentiu, South Sudan, following the mass vaccination campaign, found no significant increase in fetal loss among vaccinated women. However, the study had substantial limitations, including reliance on self-reported data, limited availability of documentation to verify pregnancy status and vaccination timing, and potential bias due to differential HEV exposure between vaccinated and unvaccinated groups. However, the double-blind cluster-randomized trial in Bangladesh indicated that 607 women who received HEV239 during pregnancy or within ninety days before pregnancy demonstrated a two-fold increased risk of spontaneous abortion.

### Added value of this study

Building upon previous findings of an elevated risk of spontaneous abortion following HEV239 vaccination, we conducted an extended analysis of the double-blind, cluster-randomized trial in Bangladesh to investigate whether maternal risk factors, spontaneous abortion characteristics, or timing of vaccination could provide insights into the mechanism behind the observed increased risk in HEV239 recipients. Our findings suggest that baseline maternal risk factors commonly associated with spontaneous abortion, such as maternal age, BMI, and histories of spontaneous abortion, hypertension, or diabetes, are unlikely to explain the elevated risk in HEV239 recipients. Additionally, the similarity in gestational age at the time of spontaneous abortion between the two groups suggests that HEV239 did not accelerate the timing of spontaneous abortion. We also found no evidence of enhanced short-term adverse reactions following HEV239 vaccination, which could indicate an inflammatory mechanism behind the observed increase in spontaneous abortion risk. However, our analysis revealed that the risk of spontaneous abortion was higher in HEV239 recipients during a critical window—specifically up to 60 days before the last menstrual period—without excess risk observed beyond this period. This finding suggests that exposure closer to conception may increase the risk of spontaneous abortion, highlighting a potential time-dependent effect that warrants further investigation.

### Implications of all the available evidence

While our analyses do not provide clues to an underlying mechanism, they do not refute the initial epidemiological findings. They instead underscore the need for more targeted biological data, such as placental examinations. Future studies should focus on exploring immune-mediated changes, particularly in aborted fetuses or placental tissues, to better understand potential mechanisms.

## Supporting information

S1 Fig Weekly predicted risk of spontaneous abortion (SAB) by time from vaccination to LMP, based on a spline-interaction model. (PNG)

S1 Table Baseline variables affecting the risk s for spontaneous abortion (SAB) among women whose zero time (ZT) occurred during LMP to −30 days. (DOCX)

S2 Table Baseline variables affecting the risk for spontaneous abortion (SAB) among women whose zero time (ZT) occurred during −31 to −60 days from LMP. (DOCX)

S3 Table Baseline variables affecting the risk for spontaneous abortion (SAB) among women whose zero time (ZT) occurred during −61 to −90 days from LMP. (DOCX)

S4 Table Baseline variables affecting the risk for spontaneous abortion (SAB) among women whose zero time (ZT) occurred during −91 to −120 days from LMP. (DOCX)

S5 Table Baseline variables affecting the risk for spontaneous abortion (SAB) among women whose zero time (ZT) occurred during −121 to −150 days from LMP. (DOCX)

S6 Table Baseline factors affecting the observed risk of spontaneous abortion (SAB) among women whose zero time (ZT) occurred during −150 to −180 days from LMP. (DOCX)
