# Supplementary figures and images for: Risk factors for spontaneous abortion following hepatitis E vaccination during and shortly before pregnancy: Further analysis from a cluster-randomized trial
Source: PLoS One. 2026 Apr 10;21(4):e0345974. doi: 10.1371/journal.pone.0345974 (PMC13068265; doi:10.1371/journal.pone.0345974)

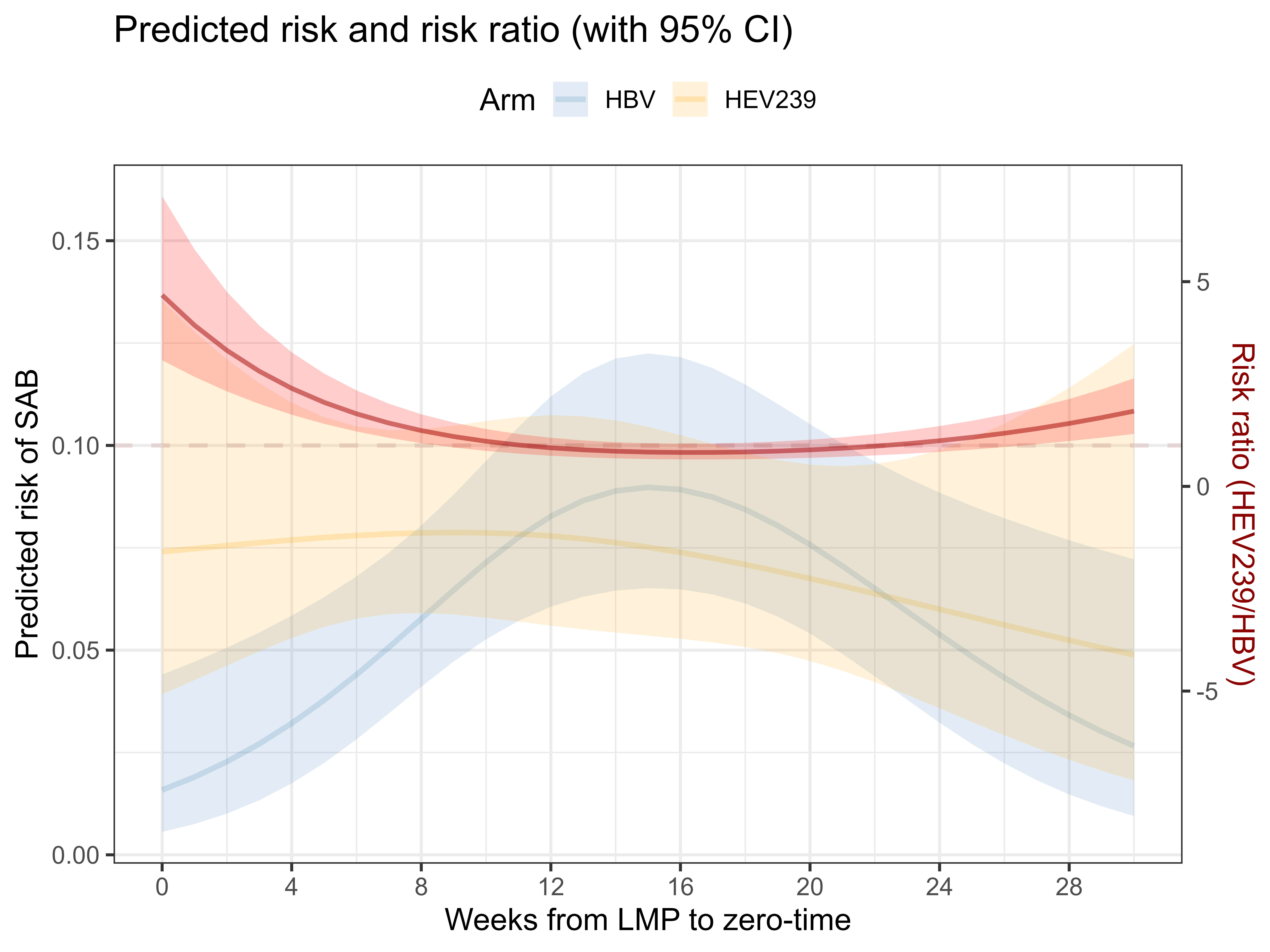

Supplement: S1 Fig — (PNG) [file pone.0345974.s001.png]
