# Supplementary material for: Risk factors for spontaneous abortion following hepatitis E vaccination during and shortly before pregnancy: Further analysis from a cluster-randomized trial
Source: PLoS One. 2026 Apr 10;21(4):e0345974. doi: 10.1371/journal.pone.0345974 (PMC13068265; doi:10.1371/journal.pone.0345974)
Supplement: S1 Table — (DOCX) [file pone.0345974.s002.docx]

**S1 Table : Baseline variables affecting the risk for spontaneous abortion (SAB) among women whose zero time (ZT) occurred during LMP to -30 days**

| **Characteristic** | **HEV239**, N = 135^1^ | **HBV**, N = 135^1^ | **p-value**^2^ |
| --- | --- | --- | --- |
| **Maternal age at ZT (Median, IQ Range)** | 24.0 (19.0, 28.0) | 24.0 (20.5, 28.0) | 0.535 |
| **Maternal age group at ZT** |  |  | 0.356 |
| 16-19, years | 36 (26.7%) | 27 (20.0%) |  |
| 20-35, years | 95 (70.4%) | 105 (77.8%) |  |
| 36-40, years | 4 (3.0%) | 3 (2.2%) |  |
| **Maternal age at 1st pregnancy test (Median, IQ Range)** | 24.0 (19.0, 28.0) | 24.0 (21.0, 28.0) | 0.480 |
| **Maternal age group at 1st pregnancy test** |  |  | 0.240 |
| 16-19, years | 35 (25.9%) | 25 (18.5%) |  |
| 20-35, years | 95 (70.4%) | 107 (79.3%) |  |
| 36-40, years | 5 (3.7%) | 3 (2.2%) |  |
| **Time difference between LMP (in days) and vaccination (Median, IQ Range)** | -14 (-23, -7) | -17 (-25, -8) | 0.121 |
| **Time difference between LMP (in weeks) and vaccination (Median, IQ Range)** |  |  | 0.361 |
| -7,-4, weeks | 39 (28.9%) | 47 (34.8%) |  |
| -3,-0, weeks | 96 (71.1%) | 88 (65.2%) |  |
| **Gestational age at first positive pregnancy test (Median, IQ Range)** | 11.0 (8.0, 16.0) | 11.0 (8.0, 14.5) | 0.457 |
| **Gestational age group at first positive pregnancy test** |  |  | 0.648 |
| 0-3, weeks | 1 (0.7%) | 1 (0.7%) |  |
| 4-6, weeks | 13 (9.6%) | 11 (8.1%) |  |
| 7-10, weeks | 46 (34.1%) | 50 (37.0%) |  |
| 11-13, weeks | 23 (17.0%) | 34 (25.2%) |  |
| 14-16, weeks | 20 (14.8%) | 14 (10.4%) |  |
| 17-19, weeks | 16 (11.9%) | 15 (11.1%) |  |
| 20-39, weeks | 16 (11.9%) | 10 (7.4%) |  |
| **BMI at enrollment (Median, IQ Range)** | 22.4 (20.1, 25.0) | 22.5 (20.6, 25.4) | 0.554 |
| **BMI group at enrollment (dose 1)** |  |  | >0.999 |
| <=30 | 131 (97.0%) | 130 (96.3%) |  |
| >30 | 4 (3.0%) | 5 (3.7%) |  |
| **History of SAB (dose 1)** |  |  | 0.392 |
| Yes | 10 (7.4%) | 14 (10.4%) |  |
| No | 125 (92.6%) | 121 (89.6%) |  |
| **History of induced /therapeutic abortion (dose 1)** |  |  | >0.999 |
| Yes | 5 (3.7%) | 4 (3.0%) |  |
| No | 130 (96.3%) | 131 (97.0%) |  |
| **History of hypertension (dose 1)** |  |  | 0.622 |
| Yes | 1 (0.7%) | 3 (2.2%) |  |
| No | 133 (98.5%) | 132 (97.8%) |  |
| **Parity** |  |  | 0.793 |
| 0 | 44 (32.6%) | 41 (30.4%) |  |
| >=1 | 91 (67.4%) | 94 (69.6%) |  |
| **History of stillbirth** |  |  | 0.722 |
| Yes | 3 (2.2%) | 5 (3.7%) |  |
| No | 132 (97.8%) | 130 (96.3%) |  |
| **History of Diabetes** |  |  | >0.999 |
| Yes | 1 (0.7%) | 1 (0.7%) |  |
| No | 133 (98.5%) | 134 (99.3%) |  |

^1^n (%); Median (IQR)

^2^Fisher's exact test; Pearson's Chi-squared test; Wilcoxon rank sum test
