# Supplementary material for: Risk factors for spontaneous abortion following hepatitis E vaccination during and shortly before pregnancy: Further analysis from a cluster-randomized trial
Source: PLoS One. 2026 Apr 10;21(4):e0345974. doi: 10.1371/journal.pone.0345974 (PMC13068265; doi:10.1371/journal.pone.0345974)
Supplement: S2 Table — (DOCX) [file pone.0345974.s003.docx]

**S2 Table : Baseline variables affecting the risk for spontaneous abortion (SAB) among women whose zero time (ZT) occurred during -31 to -60 days from LMP**

| **Characteristic** | **HEV239**, N = 145^1^ | **HBV**, N = 179^1^ | **p-value**^2^ |
| --- | --- | --- | --- |
| **Maternal age at ZT (Median, IQ Range)** | 24.0 (19.0, 29.0) | 23.0 (20.0, 27.0) | 0.306 |
| **Maternal age group at ZT** |  |  | 0.637 |
| 16-19, years | 37 (25.5%) | 38 (21.2%) |  |
| 20-35, years | 104 (71.7%) | 136 (76.0%) |  |
| 36-40, years | 4 (2.8%) | 5 (2.8%) |  |
| **Maternal age at 1st pregnancy test (Median, IQ Range)** | 24.0 (20.0, 29.0) | 23.0 (20.0, 27.0) | 0.340 |
| **Maternal age group at 1st pregnancy test** |  |  | 0.396 |
| 16-19, years | 35 (24.1%) | 33 (18.4%) |  |
| 20-35, years | 105 (72.4%) | 141 (78.8%) |  |
| 36-40, years | 5 (3.4%) | 5 (2.8%) |  |
| **Time difference between LMP (in days) and vaccination (Median, IQ Range)** | -47 (-52, -40) | -44 (-53, -37) | 0.448 |
| **Time difference between LMP (in weeks) and vaccination (Median, IQ Range)** |  |  | 0.452 |
| **Time difference between LMP (in weeks) and vaccination (Median, IQ Range)** |  |  | >0.999 |
| -11,-8, weeks | 53 (36.6%) | 65 (36.3%) |  |
| -7,-4, weeks | 92 (63.4%) | 114 (63.7%) |  |
| **Gestational age at first positive pregnancy test (Median, IQ Range)** | 11.0 (8.0, 15.0) | 10.0 (8.0, 14.0) | 0.612 |
| **Gestational age group at first positive pregnancy test** |  |  | 0.225 |
| 0-3, weeks | 1 (0.7%) | 0 (0.0%) |  |
| 4-6, weeks | 16 (11.0%) | 13 (7.3%) |  |
| 7-10, weeks | 55 (37.9%) | 80 (44.7%) |  |
| 11-13, weeks | 20 (13.8%) | 35 (19.6%) |  |
| 14-16, weeks | 30 (20.7%) | 34 (19.0%) |  |
| 17-19, weeks | 9 (6.2%) | 8 (4.5%) |  |
| 20-39, weeks | 14 (9.7%) | 9 (5.0%) |  |
| **BMI at enrollment (Median, IQ Range)** | 22.2 (19.4, 25.3) | 22.7 (20.2, 25.4) | 0.328 |
| **BMI group at enrollment** |  |  | >0.999 |
| <=30 | 141 (97.2%) | 174 (97.2%) |  |
| >30 | 4 (2.8%) | 5 (2.8%) |  |
| **History of SAB** |  |  | 0.234 |
| Yes | 6 (4.1%) | 13 (7.3%) |  |
| No | 139 (95.9%) | 166 (92.7%) |  |
| **History of induced /therapeutic abortion** |  |  | 0.413 |
| Yes | 4 (2.8%) | 2 (1.1%) |  |
| No | 141 (97.2%) | 177 (98.9%) |  |
| **History of hypertension** |  |  | 0.579 |
| Yes | 2 (1.4%) | 1 (0.6%) |  |
| No | 133 (91.7%) | 178 (99.4%) |  |
| Unknown | 10 (6.9%) | 0 (0.0%) |  |
| **Parity** |  |  | 0.760 |
| 0 | 56 (38.6%) | 69 (38.5%) |  |
| >=1 | 88 (60.7%) | 110 (61.5%) |  |
| Unknown | 1 (0.7%) | 0 (0.0%) |  |
| **History of stillbirth** |  |  | 0.146 |
| Yes | 6 (4.1%) | 2 (1.1%) |  |
| No | 139 (95.9%) | 177 (98.9%) |  |
| **History of Diabetes** |  |  | 0.581 |
| Yes | 2 (1.4%) | 1 (0.6%) |  |
| No | 135 (93.1%) | 178 (99.4%) |  |
| Unknown | 8 (5.5%) | 0 (0.0%) |  |
| ^1^n (%); Median (IQR) | | | |
| ^2^Fisher's exact test; Pearson's Chi-squared test; Wilcoxon rank sum test | | | |
