# Supplementary material for: Risk factors for spontaneous abortion following hepatitis E vaccination during and shortly before pregnancy: Further analysis from a cluster-randomized trial
Source: PLoS One. 2026 Apr 10;21(4):e0345974. doi: 10.1371/journal.pone.0345974 (PMC13068265; doi:10.1371/journal.pone.0345974)
Supplement: S3 Table — (DOCX) [file pone.0345974.s004.docx]

**S3 Table: Baseline variables affecting the risk for spontaneous abortion (SAB) among women whose zero time (ZT) occurred during -61 to -90 days from LMP**

| **Characteristic** | **HEV239**, N = 156^1^ | **HBV**, N = 170^1^ | **p-value**^2^ |
| --- | --- | --- | --- |
| **Number of doses before fetal lose/delivery** |  |  | >0.999 |
| **Maternal age at ZT (Median, IQ Range)** | 24.0 (20.0, 28.0) | 24.5 (20.0, 29.0) | 0.530 |
| **Maternal age group at ZT** |  |  | 0.887 |
| 16-19, years | 35 (22.4%) | 40 (23.5%) |  |
| 20-35, years | 115 (73.7%) | 125 (73.5%) |  |
| 36-40, years | 6 (3.8%) | 5 (2.9%) |  |
| **Maternal age at 1st pregnancy test (Median, IQ Range)** | 24.0 (20.0, 28.2) | 25.0 (20.0, 29.0) | 0.592 |
| **Maternal age group at 1st pregnancy test** |  |  | 0.963 |
| 16-19, years | 33 (21.2%) | 34 (20.0%) |  |
| 20-35, years | 117 (75.0%) | 129 (75.9%) |  |
| 36-40, years | 6 (3.8%) | 7 (4.1%) |  |
| **Time difference between LMP (in days) and vaccination (Median, IQ Range)** | -75 (-83, -68) | -74 (-82, -67) | 0.559 |
| **Time difference between LMP (in weeks) and vaccination (Median, IQ Range)** |  |  | 0.900 |
| -25,-12, weeks | 67 (42.9%) | 66 (38.8%) |  |
| -11,-8, weeks | 89 (57.1%) | 104 (61.2%) |  |
| **Gestational age at first positive pregnancy test (Median, IQ Range)** | 10.0 (8.0, 12.0) | 10.0 (8.0, 13.0) | 0.119 |
| **Gestational age group at first positive pregnancy test** |  |  | 0.218 |
| 0-3, weeks | 0 (0.0%) | 0 (0.0%) |  |
| 4-6, weeks | 24 (15.4%) | 19 (11.2%) |  |
| 7-10, weeks | 70 (44.9%) | 70 (41.2%) |  |
| 11-13, weeks | 38 (24.4%) | 41 (24.1%) |  |
| 14-16, weeks | 7 (4.5%) | 22 (12.9%) |  |
| 17-19, weeks | 9 (5.8%) | 6 (3.5%) |  |
| 20-39, weeks | 8 (5.1%) | 12 (7.1%) |  |
| **BMI at enrollment (Median, IQ Range)** | 23.0 (20.0, 25.5) | 23.0 (19.8, 26.1) | 0.783 |
| **BMI group at enrollment (dose 1)** |  |  | >0.999 |
| <=30 | 151 (96.8%) | 165 (97.1%) |  |
| >30 | 5 (3.2%) | 5 (2.9%) |  |
| **History of SAB** |  |  | 0.603 |
| Yes | 13 (8.3%) | 17 (10.0%) |  |
| No | 143 (91.7%) | 153 (90.0%) |  |
| **History of induced /therapeutic abortion** |  |  | 0.673 |
| Yes | 3 (1.9%) | 2 (1.2%) |  |
| No | 153 (98.1%) | 168 (98.8%) |  |
| **History of hypertension** |  |  | 0.601 |
| Yes | 2 (1.3%) | 1 (0.6%) |  |
| No | 147 (94.2%) | 169 (99.4%) |  |
| Unknown | 7 (4.5%) | 0 (0.0%) |  |
| **Parity** |  |  | 0.807 |
| 0 | 43 (27.6%) | 49 (28.8%) |  |
| >=1 | 113 (72.4%) | 121 (71.2%) |  |
| Unknown | 0 (0.0%) | 0 (0.0%) |  |
| **History of stillbirth** |  |  | 0.871 |
| Yes | 5 (3.2%) | 6 (3.5%) |  |
| No | 151 (96.8%) | 164 (96.5%) |  |
| **History of Diabetes** |  |  | - |
| Yes | 0 (0.0%) | 0 (0.0%) |  |
| No | 148 (94.9%) | 170 (100.0%) |  |
| Unknown | 8 (5.1%) | 0 (0.0%) |  |
| ^1^n (%); Median (IQR) | | | |
| ^2^Fisher's exact test; Wilcoxon rank sum test; Pearson's Chi-squared test | | | |
