# Supplementary material for: Risk factors for spontaneous abortion following hepatitis E vaccination during and shortly before pregnancy: Further analysis from a cluster-randomized trial
Source: PLoS One. 2026 Apr 10;21(4):e0345974. doi: 10.1371/journal.pone.0345974 (PMC13068265; doi:10.1371/journal.pone.0345974)
Supplement: S4 Table — (DOCX) [file pone.0345974.s005.docx]

**S4 Table: Baseline variables affecting the risk for spontaneous abortion (SAB) among women whose zero time (ZT) occurred during -91 to -120 days from LMP**

| **Characteristic** | **HEV239**, N = 162^1^ | **HBV**, N = 167^1^ | **p-value**^2^ |
| --- | --- | --- | --- |
| **Maternal age at ZT (Median, IQ Range)** | 24 (20, 29) | 24 (20, 30) | 0.381 |
| **Maternal age group at ZT** |  |  | 0.799 |
| 16-19, years | 39 (24.1%) | 38 (22.8%) |  |
| 20-35, years | 115 (71.0%) | 118 (70.7%) |  |
| 36-40, years | 8 (4.9%) | 11 (6.6%) |  |
| **Maternal age at 1st pregnancy test (Median, IQ Range)** | 24.0 (20.0, 29.0) | 24.0 (20.0, 30.0) | 0.433 |
| **Maternal age group at 1st pregnancy test** |  |  | 0.567 |
| 16-19, years | 35 (21.6%) | 34 (20.4%) |  |
| 20-35, years | 119 (73.5%) | 120 (71.9%) |  |
| 36-40, years | 8 (4.9%) | 13 (7.8%) |  |
| **Time difference between LMP (in days) and vaccination (Median, IQ Range)** | -104 (-111, -97) | -104 (-111, -97) | 0.690 |
| **Time difference between LMP (in weeks) and vaccination (Median, IQ Range)** |  |  | >0.999 |
| -25,-12, weeks | 162 (100.0%) | 167 (100.0%) |  |
| **Gestational age at first positive pregnancy test (Median, IQ Range)** | 9.0 (7.0, 13.0) | 8.0 (6.0, 11.0) | 0.035 |
| **Gestational age group at first positive pregnancy test** |  |  | 0.035 |
| 0-3, weeks | 3 (1.9%) | 0 (0.0%) |  |
| 4-6, weeks | 32 (19.8%) | 45 (26.9%) |  |
| 7-10, weeks | 65 (40.1%) | 73 (43.7%) |  |
| 11-13, weeks | 27 (16.7%) | 26 (15.6%) |  |
| 14-16, weeks | 13 (8.0%) | 12 (7.2%) |  |
| 17-19, weeks | 12 (7.4%) | 8 (4.8%) |  |
| 20-39, weeks | 10 (6.2%) | 3 (1.8%) |  |
| **BMI at enrollment (Median, IQ Range)** | 21.9 (19.4, 25.8) | 22.1 (19.8, 25.4) | 0.790 |
| **BMI group at enrollment** |  |  | 0.437 |
| <=30 | 151 (93.2%) | 159 (95.2%) |  |
| >30 | 11 (6.8%) | 8 (4.8%) |  |
| **History of SAB** |  |  | 0.913 |
| Yes | 14 (8.6%) | 15 (9.0%) |  |
| No | 148 (91.4%) | 152 (91.0%) |  |
| **History of induced /therapeutic abortion** |  |  | 0.337 |
| Yes | 3 (1.9%) | 7 (4.2%) |  |
| No | 159 (98.1%) | 160 (95.8%) |  |
| **History of hypertension in pregnancy** |  |  | 0.686 |
| Yes | 2 (1.2%) | 4 (2.4%) |  |
| No | 153 (94.4%) | 163 (97.6%) |  |
| Unknown | 7 (4.3%) | 0 (0.0%) |  |
| **Parity** |  |  | 0.353 |
| 0 | 60 (37.0%) | 53 (31.7%) |  |
| >=1 | 102 (63.0%) | 114 (68.3%) |  |
| Unknown | 0 (0.0%) | 0 (0.0%) |  |
| **History of stillbirth** |  |  | 0.215 |
| Yes | 1 (0.6%) | 5 (3.0%) |  |
| No | 161 (99.4%) | 162 (97.0%) |  |
| **History of Diabetes** |  |  | - |
| Yes | 0 (0.0%) | 0 (0.0%) |  |
| No | 154 (95.1%) | 166 (99.4%) |  |
| Unknown | 8 (4.9%) | 1 (0.6%) |  |
| ^1^n (%); Median (IQR) | | | |
| ^2^Fisher's exact test; Wilcoxon rank sum test; Pearson's Chi-squared test | | | |
