# Supplementary material for: Risk factors for spontaneous abortion following hepatitis E vaccination during and shortly before pregnancy: Further analysis from a cluster-randomized trial
Source: PLoS One. 2026 Apr 10;21(4):e0345974. doi: 10.1371/journal.pone.0345974 (PMC13068265; doi:10.1371/journal.pone.0345974)
Supplement: S5 Table — (DOCX) [file pone.0345974.s006.docx]

**S5 Table: Baseline variables affecting the risk for spontaneous abortion (SAB) among women whose zero time (ZT) occurred during -121 to -150 days from LMP**

| **Characteristic** | **HEV239**, N = 89^1^ | **HBV**, N = 98^1^ | **p-value**^2^ |
| --- | --- | --- | --- |
| **Maternal age at ZT (Median, IQ Range)** | 24.0 (20.0, 27.0) | 22.5 (20.0, 29.0) | 0.647 |
| **Maternal age group at ZT** |  |  | 0.465 |
| 16-19, years | 20 (22.5%) | 24 (24.5%) |  |
| 20-35, years | 68 (76.4%) | 70 (71.4%) |  |
| 36-40, years | 1 (1.1%) | 4 (4.1%) |  |
| **Maternal age at 1st pregnancy test (Median, IQ Range)** | 24.0 (20.0, 28.0) | 23.0 (20.0, 30.0) | 0.754 |
| **Maternal age group at 1st pregnancy test** |  |  | 0.847 |
| 16-19, years | 16 (18.0%) | 19 (19.4%) |  |
| 20-35, years | 70 (78.7%) | 74 (75.5%) |  |
| 36-40, years | 3 (3.4%) | 5 (5.1%) |  |
| **Time difference between LMP (in days) and vaccination (Median, IQ Range)** | -136 (-144, -129) | -132 (-141, -126) | 0.068 |
| **Time difference between LMP (in weeks) and vaccination (Median, IQ Range)** |  |  | 0.347 |
| -25,-12, weeks | 89 (100.0%) | 98 (100.0%) |  |
| **Gestational age at first positive pregnancy test (Median, IQ Range)** | 10.0 (8.0, 14.0) | 10.5 (8.0, 14.0) | 0.986 |
| **Gestational age group at first positive pregnancy test** |  |  | 0.811 |
| 0-3, weeks | 0 (0.0%) | 0 (0.0%) |  |
| 4-6, weeks | 9 (10.1%) | 6 (6.1%) |  |
| 7-10, weeks | 39 (43.8%) | 43 (43.9%) |  |
| 11-13, weeks | 14 (15.7%) | 23 (23.5%) |  |
| 14-16, weeks | 12 (13.5%) | 12 (12.2%) |  |
| 17-19, weeks | 9 (10.1%) | 7 (7.1%) |  |
| 20-39, weeks | 6 (6.7%) | 7 (7.1%) |  |
| **BMI at enrollment (Median, IQ Range)** | 22.4 (19.0, 25.7) | 22.8 (20.2, 25.6) | 0.471 |
| **BMI group at enrollment** |  |  | 0.738 |
| <=30 | 84 (94.4%) | 94 (95.9%) |  |
| >30 | 5 (5.6%) | 4 (4.1%) |  |
| **History of SAB** |  |  | 0.884 |
| Yes | 5 (5.6%) | 6 (6.1%) |  |
| No | 84 (94.4%) | 92 (93.9%) |  |
| **History of induced /therapeutic abortion** |  |  | >0.999 |
| Yes | 0 (0.0%) | 0 (0.0%) |  |
| No | 89 (100.0%) | 98 (100.0%) |  |
| **History of hypertension** |  |  | 0.225 |
| Yes | 0 (0.0%) | 1 (1.0%) |  |
| No | 87 (97.8%) | 97 (99.0%) |  |
| Unknown | 2 (2.2%) | 0 (0.0%) |  |
| **Parity** |  |  | 0.187 |
| 0 | 25 (28.1%) | 37 (37.8%) |  |
| >=1 | 63 (70.8%) | 61 (62.2%) |  |
| Unknown | 1 (1.1%) | 0 (0.0%) |  |
| **History of stillbirth** |  |  | >0.999 |
| Yes | 1 (1.1%) | 2 (2.0%) |  |
| No | 88 (98.9%) | 96 (98.0%) |  |
| **History of Diabetes** |  |  | - |
| Yes | 0 (0.0%) | 0 (0.0%) |  |
| No | 85 (95.5%) | 98 (100.0%) |  |
| Unknown | 4 (4.5%) | 0 (0.0%) |  |
| ^1^n (%); Median (IQR) | | | |
| ^2^Fisher's exact test; Wilcoxon rank sum test; Pearson's Chi-squared test | | | |
