# Supplementary material for: Risk factors for spontaneous abortion following hepatitis E vaccination during and shortly before pregnancy: Further analysis from a cluster-randomized trial
Source: PLoS One. 2026 Apr 10;21(4):e0345974. doi: 10.1371/journal.pone.0345974 (PMC13068265; doi:10.1371/journal.pone.0345974)
Supplement: S6 Table — (DOCX) [file pone.0345974.s007.docx]

**S6 Table: Baseline factors affecting the risk for spontaneous abortion (SAB) among women whose zero time (ZT) occurred during -150 to -180 days from LMP**

| **Characteristic** | **HEV239**, N = 72^1^ | **HBV**, N = 85^1^ | **p-value**^2^ |
| --- | --- | --- | --- |
| **Maternal age at ZT (Median, IQ Range)** | 23.0 (18.0, 28.0) | 24.0 (20.0, 30.0) | 0.115 |
| **Maternal age group at ZT** |  |  | 0.192 |
| 16-19, years | 23 (31.9%) | 20 (23.5%) |  |
| 20-35, years | 49 (68.1%) | 62 (72.9%) |  |
| 36-40, years | 0 (0.0%) | 3 (3.5%) |  |
| **Maternal age at 1st pregnancy test (Median, IQ Range)** | 24.0 (19.0, 28.2) | 25.0 (21.0, 31.0) | 0.145 |
| **Maternal age group at 1st pregnancy test** |  |  | 0.035 |
| 16-19, years | 19 (26.4%) | 15 (17.6%) |  |
| 20-35, years | 53 (73.6%) | 64 (75.3%) |  |
| 36-40, years | 0 (0.0%) | 6 (7.1%) |  |
| **Time difference between LMP (in days) and vaccination (Median, IQ Range)** | -169 (-174, -160) | -166 (-171, -157) | 0.056 |
| **Time difference between LMP (in weeks) and vaccination (Median, IQ Range)** |  |  | 0.338 |
| -52,-27, weeks | 14 (19.4%) | 9 (10.6%) |  |
| -25,-12, weeks | 58 (80.6%) | 76 (89.4%) |  |
| **Gestational age at first positive pregnancy test (Median, IQ Range)** | 11.0 (9.0, 14.0) | 10.0 (7.0, 13.0) | 0.047 |
| **Gestational age group at first positive pregnancy test** |  |  | 0.363 |
| 0-3, weeks | 0 (0.0%) | 0 (0.0%) |  |
| 4-6, weeks | 4 (5.6%) | 10 (11.8%) |  |
| 7-10, weeks | 29 (40.3%) | 38 (44.7%) |  |
| 11-13, weeks | 18 (25.0%) | 17 (20.0%) |  |
| 14-16, weeks | 9 (12.5%) | 12 (14.1%) |  |
| 17-19, weeks | 5 (6.9%) | 2 (2.4%) |  |
| 20-39, weeks | 7 (9.7%) | 6 (7.1%) |  |
| **BMI at enrollment (Median, IQ Range)** | 21.9 (19.3, 25.0) | 21.4 (19.0, 24.5) | 0.505 |
| **BMI group at enrollment** |  |  | >0.999 |
| <=30 | 67 (93.1%) | 80 (94.1%) |  |
| >30 | 5 (6.9%) | 5 (5.9%) |  |
| **History of SAB** |  |  | 0.471 |
| Yes | 5 (6.9%) | 3 (3.5%) |  |
| No | 67 (93.1%) | 82 (96.5%) |  |
| **History of induced /therapeutic abortion** |  |  | >0.999 |
| Yes | 1 (1.4%) | 1 (1.2%) |  |
| No | 71 (98.6%) | 84 (98.8%) |  |
| **History of hypertension in pregnancy** |  |  | 0.167 |
| Yes | 0 (0.0%) | 3 (3.5%) |  |
| No | 71 (98.6%) | 82 (96.5%) |  |
| Unknown | 1 (1.4%) | 0 (0.0%) |  |
| **Parity** |  |  | 0.747 |
| 0 | 32 (44.4%) | 35 (41.2%) |  |
| >=1 | 40 (55.6%) | 50 (58.8%) |  |
| Unknown | 0 (0.0%) | 0 (0.0%) |  |
| **History of stillbirth** |  |  | >0.999 |
| Yes | 1 (1.4%) | 1 (1.2%) |  |
| No | 71 (98.6%) | 84 (98.8%) |  |
| **History of Diabetes** |  |  | 0.708 |
| Yes | 0 (0.0%) | 1 (1.2%) |  |
| No | 71 (98.6%) | 84 (98.8%) |  |
| Unknown | 1 (1.4%) | 0 (0.0%) |  |
| ^1^n (%); Median (IQR) | | | |
| ^2^Fisher's exact test; Wilcoxon rank sum test; Pearson's Chi-squared test | | | |
